# Lin28A activates androgen receptor via regulation of c-myc and promotes malignancy of ER−/Her2+ breast cancer

**DOI:** 10.18632/oncotarget.11004

**Published:** 2016-08-02

**Authors:** Honghong Shen, Lin Zhao, Xiaolong Feng, Cong Xu, Congying Li, Yun Niu

**Affiliations:** ^1^ Tianjin Medical University Cancer Institute and Hospital, National Clinical Research Center for Cancer, Key Laboratory of Cancer Prevention and Therapy of Tianjin, Key Laboratory of Breast Cancer Prevention and Therapy, Tianjin Medical University, Ministry of Education, Ti Yuan Bei, Tianjin 300060, People's Republic of China

**Keywords:** breast cancer, Lin28A, androgen receptor, c-myc, Her2

## Abstract

Having previously demonstrated the co-expression status of the Lin28A and androgen receptor (AR) in ER−/Her2+ breast cancer, we tested the hypothesis that Lin28A can activate AR and promotes growth of ER−/Her2+ breast cancer. The expression of Lin28A and AR were examined after Lin28A siRNA and Lin28A plasmid were transfected into ER−/Her2+ breast cancer cells. Chromatin immune-precipitation (ChIP) analysis and Luciferase Assays were used to evaluate the effect of Lin28A and c-myc on AR promoter activity. MTT assays, Boyden chamber invasion assays, colony formation assays and flow cytometry analysis were performed. ER−/Her2+ breast cancer cells which transfected with Lin28A siRNAs and Lin28A plasmid were injected into nude mice, and tumorigenesis was monitored. Our data showed that Lin28A can induced AR expression in ER−/Her2+ breast cancer cells. ChIP analysis showed that Lin28A stimulates the recruitment of c-Myc to the promoter of the AR gene. Lin28A enhanced growth ability, colonies ability, cells proliferation activities, invasive ability and inhibited cells apoptosis of ER−/Her2+ breast cancer cells. Lin28A high expression cells exhibited significantly higher tumorigenic ability *in vivo*. Our study demonstrates that Lin28A can activates androgen receptor via regulation of c-myc and promotes malignancy of ER−/Her2+ breast cancer. Our findings underline a novel role for Lin28A in breast cancer development and activation of the AR axis.

## INTRODUCTION

Sex steroid hormones are critical for the development and progression of breast cancer. Estrogen is widely recognized for its role in breast cancer, while little is known about the potential role for androgen in this disease. Endocrine therapies that target estrogen and estrogen receptor (ER) have led to significant progress in the treatment and prevention of the majority of breast cancer patients. While in contrast to ER positive(ER+) breast cancer, there are currently limited knowledge available regarding the biology of ER negative(ER−) breast tumors [1]. Therapies targeting Her2 such as trastuzumab are becoming increasingly important in the treatment of Her2+ tumors. Nevertheless, there are significant numbers of breast tumors fail to respond. Recent studies have found that androgen receptor (AR) is highly expressed in ER-/Her2+ breast tumors and androgens and AR stimulate the growth of ER−/Her2+ breast cancer cells by activating oncogenic Wnt signaling pathways [2].

In recent years, the highly conserved RNA-binding protein Lin28A has emerged as factors that increased the self-renewal of mammalian embryonic stem cells [3]. Lin28A has been shown overexpressed in diverse human malignancies [4, 5] and promoted tumorigenesis and proliferation of cancer cells [6–8]. Lin28A can increase AR expression and its target genes such as PSA and NKX3.1 and enhances growth of human CaP cells [9]. However, similar studies concerning the impact of Lin28A on expression of the AR in human breast cancer have not been reported previously. Lin28A also is a master regulator of let-7 miRNA processing, binds to the terminal loops of the precursors of let-7 family miRNAs and blocks their processing into mature miRNAs [10, 11]. Lin28A also increases c-myc by repressing let-7, and c-myc transcriptionally activates Lin28A [12].

Having previously demonstrated the co-expression status of Lin28A and androgen receptor (AR) in ER−/Her2+ breast cancer [13], this study showed that Lin28A can induce the expression of AR via regulation of c-myc in ER−/Her2+ breast cancer. We transfected Lin28A siRNA and Lin28A plasmid transiently into ER−/Her2+ breast cancer cells and found that increase of AR expression can enhances growth, invasion, and soft agar colony formation. The expression of Lin28A also promoted tumorigenicity of in nude mices which injected with ER−/Her2+ breast cancer cells. Taken together, these data demonstrated the functional importance of Lin28A and AR in human ER−/Her2+ breast cancer.

## RESULTS

### Lin28A increases the expression of AR in ER-/Her2+ breast cancer cells

**Figure 1:**
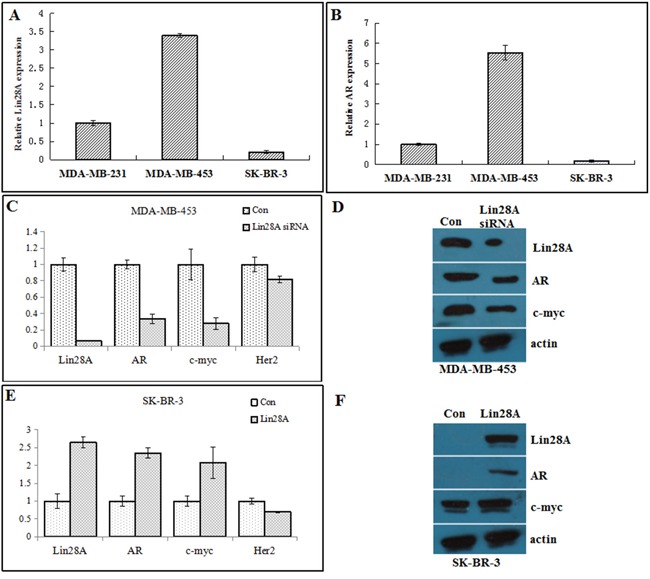
Lin28A increases AR and c-myc expression Relative expression levels of Lin28A **A.** and AR **B.** were determined in MDA-MB-231, MDA-MB-453, and SK-BR-3 cells by qRT-PCR. Results are presented as relative fold change compared to expression levels in MDA-MB-231 cells. **C.** MDA-MB-453 cells were transfected with Lin28A siRNA#2 plasmid, and AR and c-myc mRNA levels were analyzed by qRT-PCR. Results are presented as relative fold change compared to expression levels in MDA-MB-453 cells transfected with control plasmid. Con, control. **D.** Western blot analysis showing the decrease in AR and c-myc protein expression in MDA-MB-453 cells transfected with Lin28A siRNA#2 plasmid. Actin is shown as loading control. **E.** SK-BR-3 cells were transfected with Lin28A plasmid, and AR and c-myc mRNA levels were analyzed by qRT-PCR. **F.** Western blot analysis showing the up-regulation of AR and c-myc protein expression in SK-BR-3 cells transfected with Lin28A.

We initially determined Lin28A and AR expression in ER-/Her2+ and ER-/Her2- breast tumor cells lines. The level of AR were higher in the MDA-MB-453 cells (ER-/Her2+), which express higher levels of Lin28A compared with MDA-MB-231 cells (ER-/Her2-) that express lower levels of Lin28A and AR. The levels of AR were lower in the SK-BR-3 cells (ER-/Her2+), which express lower levels of Lin28A compared with MDA-MB-231 cells (Figure 1A and 1B), indicating a positive relationship between Lin28A and AR. To test whether Lin28A affects AR expression in ER-/Her2+ breast cancer cells, Lin28A siRNA#2(Lin28A siRNA#2 could obviously decrease the expression of Lin28A compared with Lin28A siRNA#1 and Lin28A siRNA#3) were transfected into MDA-MB-453, plasmid overexpressed Lin28A(Lin28A) were transfected into SK-BR-3 cells, and the expression level of AR mRNA and c-myc mRNA was analyzed by qRT-PCR. Down-regulation of Lin28A decreased AR mRNA and c-myc mRNA level 3.5-fold and 3-fold, respectively, in MDA-MB-453 cells (Figure 1C). Then we analyzed whole cell lysates from Lin28A siRNA MDA-MB-453 cells by Western blot analysis. The AR and c-myc protein level was decreased in MDA-MB-453 cells when Lin28A was down-regulated (Figure 1D). Over-expression of Lin28A increased AR mRNA and c-myc mRNA level 2.7-fold and 2-fold, respectively, in SK-BR-3 cells (Figure 1E). The AR and c-myc protein level were increased in SK-BR-3 cells when Lin28A was up-regulated (Figure 1F). Collectively, these data demonstrate that Lin28A increased AR and c-myc expression in ER-/Her2+ breast cancer cells.

### Promotion of AR by Lin28A is mediated by c-myc

**Figure 2:**
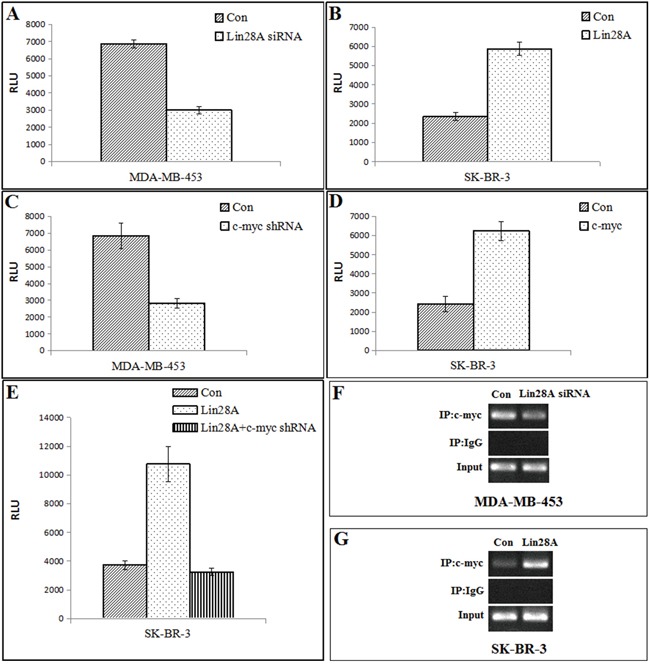
Regulation of AR by Lin28A is mediated by regulation of c-myc **A.** MDA-MB-453 cells were cotransfected with control or Lin28AsiRNA along with the pGL4-AR-Prom-Luc reporter. AR promoter activity was repressed when Lin28A was down-regulated. Data points represent the mean±S.D. of triplicate samples from two independent experiments. RLU: Relative Luciferase Units. **B.** SK-BR-3 cells were cotransfected with control or Lin28A along with the pGL4-AR-Prom-Luc reporter. AR promoter activity was enhanced in Lin28A-overexpressing cells. **C.** MDA-MB-453 cells were cotransfected with control or c-myc shRNA along with the pGL4-AR-Prom-Luc reporter. Luciferase assays showed that down-regulation of c-myc reduced transactivation of the AR promoter. **D.** SK-BR-3 cells were cotransfected with control or c-myc along with the pGL4-AR-Prom-Luc reporter. Overexpression of c-myc enhanced AR promoter activity. **E.** SK-BR-3 cells were transfected with Lin28A alone or Lin28A and c-myc shRNA together along with the pGL4-AR prom-Luc reporter. Repression of c-myc could overcome the promotion of AR promoter activity by Lin28A. **F, G.** Recruitment of c-myc to the c-myc binding site in the AR promoter was analyzed by ChIP assays. Overexpression of Lin28A enhanced the recruitment of c-myc to the AR promoter. Down-regulation of Lin28A reduced the recruitment of c-myc to the AR promoter.

To examine whether Lin28A affects transcription of AR, Lin28AsiRNA or Lin28A plasmid were cotransfected with a luciferase reporter driven by the full-length promoter of the AR gene into MDA-MB-453 cells or SK-BR-3 cells, respectively, and luciferase assays were performed. Down-regulation of Lin28A expression by Lin28AsiRNA decreased, whereas overexpression of Lin28A increased, the activation of the AR promoter (Figure 2A, 2B), suggesting that promotion of AR expression by Lin28A may be at the level of transcription.

Because c-myc, which activates AR transcription by binding to a consensus element in the AR promoter [14], was one of the targets of let-7c, and Lin28A can binds to the terminal loops of the precursors of let-7 family miRNAs and blocks their processing into mature miRNAs, we hypothesized that a let-7c target gene(c-myc) may function as a transcriptional regulator of AR. Down-regulation of c-myc by c-myc shRNA reduced AR promoter activity (Figure 2C), whereas overexpression of c-myc enhanced AR promoter activity (Figure 2D).

We next cotransfected Lin28A with c-myc shRNA in SK-BR-3 cells(Lin28A negative type) expressing a luciferase reporter driven by the full-length AR promoter. The results showed that Lin28A increased AR promoter activity, which was reversed by suppression of c-myc (Figure 2E). ChIP assays were performed using primers spanning the consensus binding site for c-myc in the AR promoter to determine whether Lin28A activates the recruitment of c-myc to the promoter of the AR gene. Overexpression of Lin28A increased the recruitment of c-myc to the AR promoter (Figure 2F, 2G).

### Influence of AR expression by Lin28A affects proliferation of of ER-/Her2+ breast cancer cells *in vitro*

**Figure 3:**
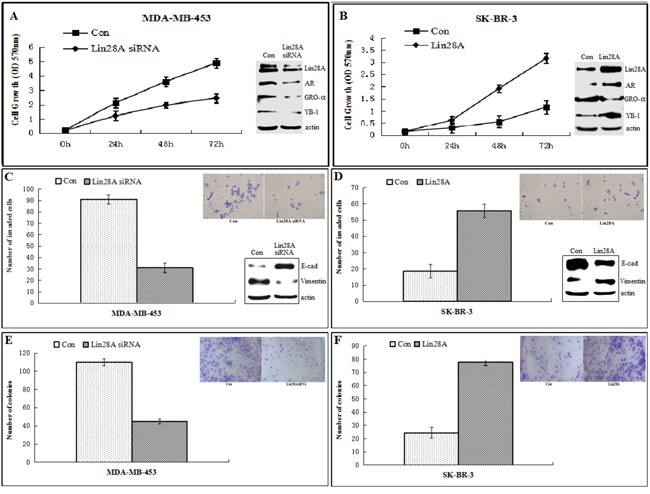
Lin28A promotes growth, invasive and clonogenic ability of ER-/Her2+ breast cancer cells **A, B.** MDA-MB-453 cells were transfected with empty vector or Lin28A siRNA#2 plasmid and SK-BR-3 cells were transfected with empty vector or Lin28A plasmid. Cells growth was monitored at 0, 24, 48, 72 hours. Expression of Lin28A enhanced growth rates of ER-/Her2+ breast cancer cells. Western blot analysis of a series of cell proliferation related molecules, including GRO-α and YB-1 were detected. **C, D.** Boyden chamber invasion assays showed that Lin28A increases invasiveness of ER-/Her2+ breast cancer cells. Inset: Representative images of invading cells. Western blot analysis of E-cadherin and Vimentin were detected. **E, F.** Lin28A-expressing ER-/Her2+ breast cancer cells exhibited higher clonogenic ability compared to control cells. Inset: Representative images of clonogenic cells. Data are presented as means±SD of three experiments performed in triplicate.

Cell growth of MDA-MB-453 and SK-BR-3 and the expression levels of cell prolification factors GRO-α and YB-1 were detected by transfecting Lin28A siRNA and Lin28A, respectively. Decreased expression of Lin28A reduced growth of MDA-MB-453 cells, and the expression of GRO-α and YB-1 was decreased in Lin28A siRNA group compared with control group (Figure 3A). Similarly, SK-BR-3 cells expressing Lin28A exhibited a higher rate of growth compared with control group, and the expression of GRO-α and YB-1 was increased in Lin28A group compared with control group (Figure 3B), indicating that suppression of AR expression by Lin28A leads to reduced survival of ER-/Her2+ breast cancer cells.

### Influence of AR expression by Lin28A increases invasiveness of ER-/Her2+ breast cancer cells *in vitro*

Cells were plated on Matrigel in the upper compartment of the Boyden chamber and allowed to invade toward the lower compartment filled with complete medium containing complete fetal bovine serum (FBS). The number of Lin28A siRNA MDA-MB-453 cells invading through Matrigel in FBS-containing medium was 31±4, whereas the number of control cells invading through Matrigel was 91±5, and the expression of E-cadherin was increased, while the expression of vimentin was decreased in Lin28A siRNA group compared with control group (Figure 3C). Similarly, the number of Lin28A SK-BR-3 cells invading through Matrigel in FBS-containing medium was 55±4, whereas the number of control cells was 18±4, and the expression of E-cadherin was decreased, while the expression of vimentin was increased in Lin28A group compared with control group (Figure 3D). Based on these results, we speculated that Lin28A induces invasion of ER-/Her2+ breast cancer cells through basement membrane *in vitro*.

### Influence of AR expression by Lin28A increases clonogenic ability of ER-/Her2+ breast cancer cells *in vitro*

Clonogenic assays showed that the number of colonies formed by Lin28A siRNA MDA-MB-453 cells was 45±5, whereas the number of colonies formed by control MDA-MB-453 cells was 110±10 (Figure 3E). Similarly, the number of colonies formed by SK-BR-3 cells expressing Lin28A was 77±5, whereas the number of colonies formed by control SK-BR-3 cells was 24±4 (Figure 3F).

### Influence of AR expression by Lin28A inhibited ER-/Her2+ breast cancer cells apoptosis

**Figure 4:**
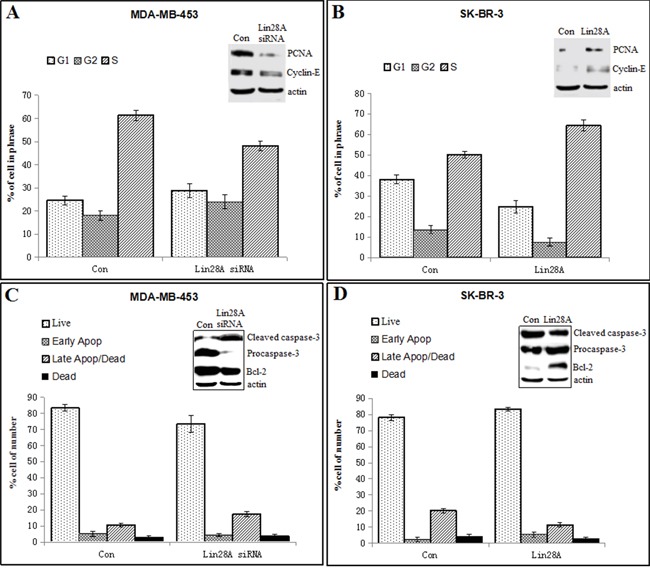
Lin28A played a critical role in the G1/S progression and apoptosis in ER-/Her2+ breast cancer cells **A.** The flow cytometry analysis showed that the number of MDA-MB-453 cells in G1 phase increased, while the number of cells in S phase decreased in the Lin28A siRNA#2 group compared to the control group. Western blot analysis of a series of cell cycle-related molecules, including PCNA and cyclin E were detected. **B.** The number of SK-BR-3 cells in G1 phase decreased, while the number of cells in S phase increased in the Lin28A group compared to the control group. Western blot analysis of a series of cell cycle-related molecules, including PCNA and cyclin E were detected. **C.** The percentage of apoptotic cells was higher in the Lin28A siRNA#2 group compared to the control group in MDA-MB-453 cells. Western blotting was used to analyse the levels of the anti-apoptosis marker Bcl-2, pro-apoptotic marker procaspase-3 and cleaved caspase-3. **D.** The percentage of apoptotic cells was lower in the Lin28A group compared to the control group in SK-BR-3 cells. Western blotting was used to analyse the levels of the anti-apoptosis marker Bcl-2, pro-apoptotic marker procaspase-3 and cleaved caspase-3.

We performed flow cytometry to analyse cell cycle and apoptosis in ER-/Her2+ breast cancer cells, and then we analysed some markers of cell cycle and apoptosis by Western blotting, including PCNA, cyclin E, cleaved caspase-3, procaspase-3 and Bcl-2. Data showed that the number of MDA-MB-453 cells in G1 phase increased, while the number of cells in S phase decreased in the Lin28A siRNA group compared to the control group, and the expression of PCNA and cyclin-E was decreased in Lin28A siRNA group compared with control group (Figure 4A). The number of SK-BR-3 cells in G1 phase decreased, while the number of cells in S phase increased in the Lin28A group compared to the control group, and the expression of PCNA and cyclin-E was increased in Lin28A group compared with control group (Figure 4B). The percentage of apoptotic cells was higher in the Lin28A siRNA group compared to the control group in MDA-MB-453 cells, and the levels of pro-apoptotic marker procaspase-3 and the anti-apoptosis marker Bcl-2 were significantly decreased and the levels of the cleaved caspase-3 were increased in Lin28A siRNA group compared with control group (Figure 4C). The percentage of apoptotic cells was lower in the Lin28A group compared to the control group in SK-BR-3 cells, and the levels of procaspase-3 and the anti-apoptosis marker Bcl-2 were increased and the levels of the cleaved caspase-3 were decreased in Lin28A group compared with control group (Figure 4D). These results indicated that Lin28A played a critical role in the G1/S progression and apoptosis in ER-/Her2+ breast cancer cells.

### Influence of AR expression by Lin28A promotes tumorigenicity of ER-/Her2+ breast tumor cells *in vivo*

**Figure 5:**
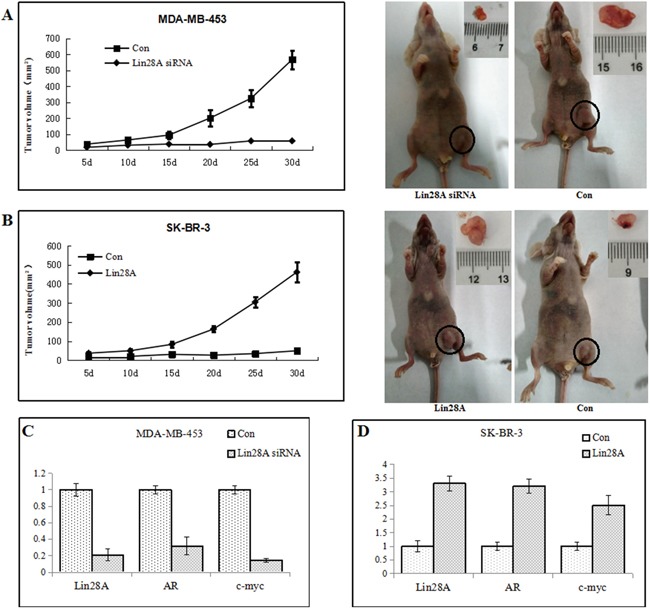
Lin28A promotes tumor growth of ER-/Her2+ breast cancer *in vivo* We injected 2×10^6^/100ul Lin28A siRNA MDA-MB-453 cells or control cells and Lin28A SK-BR-3 cells or control cells into subcutaneous of female nude mice. **A.** Mice tumors which injected with Lin28A siRNA MDA-MB-453 cells exhibited significantly lower rates of incidence and growth of tumors compared to control cells(Left: tumor growth curve graph; Right: mice and tumor). **B.** Mice tumors injected with Lin28A SK-BR-3 cells exhibited significantly higher tumors growth compared to control cells(Left: tumor growth curve graph; Right: mice and tumor). **C, D.** Total RNA extracts from the tumors were examined for expression levels of Lin28A and AR. Lin28A was correlated positively with expression levels of AR in ER-/Her2+ breast tumor.

Twenty nude mice were randomly divided into four groups. We injected 2×10^6^/100ul Lin28A siRNA MDA-MB-453 cells or control cells and Lin28A SK-BR-3 cells or control cells into subcutaneous of female nude mice. Tumors were measured every 5 days. We found that mices injected with Lin28A siRNA MDA-MB-453 cells exhibited significantly lower rates tumor growth compared to control cells (Figure 5A). Mices injected with Lin28A SK-BR-3 cells exhibited significantly higher tumors growth compared to control cells (Figure 5B). Total RNA extracts from the tumors were examined for expression levels of Lin28A and AR. The results indicated that higher expression of Lin28A may enhances expression of AR and promotes tumor growth of ER-/Her2+ breast tumor cells via regulation of c-myc *in vivo*.

**Figure 6:**
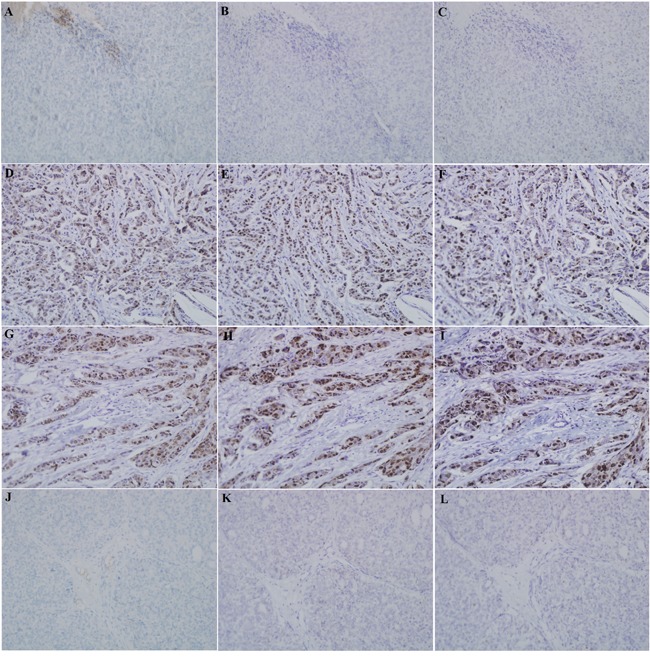
The expression of Lin28A, AR, and Ki67 in xenograft tumor was detected by using immunohistochemistry Breast cancer cells were inoculated in the left inguinal mammary fat pad of syngeneic Balb/c mice, and the mice were divided into four groups. In mice tumors which injected with MDA-MB-453 cells, Lin28A siRNA group showed downregulation of Lin28A **A.**, AR **B.** and Ki67 **C.** expression compared with the control group **D, E, F.** In mice tumors which injected with SK-BR-3 cells, Lin28A group induced upregulation of Lin28A **G.**, AR **H.** and Ki67 **I.** expression compared to the control group **J, K, L.**

**Figure 7:**
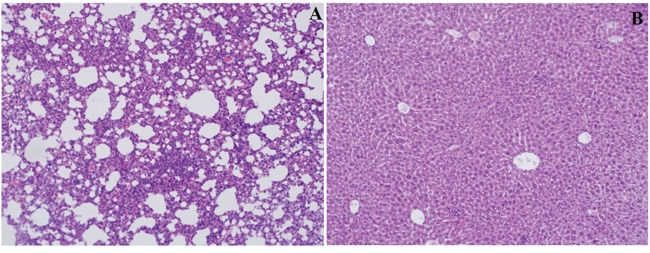
Hematoxylin-eosin staining of mice lungs A. and livers B

The expression of Lin28A, AR, and Ki67 in xenograft tumor was detected by using immunohistochemistry. In mice tumors which injected with MDA-MB-453 cells, Lin28A siRNA group showed decreased expression of Lin28A (Figure 6A), AR (Figure 6B) and Ki67 (Figure 6C) compared with the control group (Figure 6D, 6E, 6F). In mice tumors which injected with SK-BR-3 cells, Lin28A group showed increased expression of Lin28A (Figure 6G), AR (Figure 6H) and Ki67 (Figure 6I) compared to the control group (Figure 6J, 6K, 6L). While there was no metastasis found in lungs and livers among all groups (Figure 7A, 7B).

## DISCUSSION

In this study, we found that Lin28A can activates androgen receptor(AR) via regulation of c-myc and promote ER-/Her2+ breast tumor growth. Endocrine therapies that target estrogen and ER signaling pathways play a critical role in the treatment of the majority of breast cancer patients [15]. However, over a quarter of breast tumors fail to express ER and are thus resistant to these therapies [16]. Androgen receptor (AR) is expressed in 60-70% of breast tumors, independent of ER status [17–19]. Our findings suggest that there are a significant number of ER−/Her2+ breast tumors express Lin28A and AR and Lin28A can activate AR expression.

It is well documented that Lin28A can increase let-7c expression [11, 20]. Overexpression of Lin28A inhibited let-7c expression, whereas knockdown of Lin28A increased let-7c expression in breast cancer cells [12, 21]. The let-7 family miRNAs are tumor suppressors and are implicated as prognostic factors in a multitude of cancers. The most important and well studied targets of let-7 are the oncogenes: HMGA2, RAS, and Myc. Each of these proteins is an important transcription factor, and it is conceivable that by repressing their expression directly [22, 23]. Recent evidence suggests that Lin28A does not rely solely on its regulation of let-7 miRNA biogenesis, but also modulates gene expression by altering translation [24–26]. Lin28 plays an important role in promoting c-myc-dependent cellular proliferation [3]. Lin28A may regulate expression of prosurvival genes in ER-/Her2+ breast cancer via one of these mechanisms. In our study, overexpression of Lin28A introduced AR and c-myc expression, whereas knockdown of Lin28A inhibited AR and c-myc expression in ER-/Her2+ breast cancer cells. Lin28A introduces AR expression and activity in ER-/Her2+ breast cancer cells by targeting its transcription via c-myc. In addition, a positive correlation was found between the expression of Lin28A and AR in the tumors of ER-/Her2+ breast cancer xenograft models, indicating that a positive feedback loop exists between Lin28A and AR.

Her-2 gene amplification is long known to contribute to elevated Her-2 protein levels in breast and other cancers [27–28]. Her-2 directed antibody trastuzumab has increasingly been exploited for treating cancers other than breast cancer [29]. However, a substantial proportion of breast cancer patients do not respond to this drug, Targeting Her-2 alone by trastuzumab may, under some conditions, not be sufficient to halt the rapid growth of tumor cells that also express high levels of Lin28A. Our results indicate that, if feasible strategies to reconstitute Lin28A in breast tumors can be developed, reconstitution of Lin28A may become a potential therapeutic agent for breast cancer treatment. The down-regulation of potential oncogenic and survival factors by Lin28A may represent an attractive therapeutic opportunity in human breast cancer. Further studies are required to elucidate the roles of Lin28A and AR network in breast cancers.

In summary, we show that Lin28A plays an important activating role in the AR expression via c-myc and thereby promotes ER-/Her2+ breast cancer cell prolification and invasiveness. Our results suggest that the expression of Lin28A may be an attractive target for therapeutic intervention by enhancing AR in ER-/Her2+ breast cancer.

## MATERIALS AND METHODS

### Cell lines and culture conditions for ER−/Her2+ breast cancer

We previously found that MDA-MB-453 and SK-BR-3 cells had over-expression of Her2 and low-expression of ER [13]. This pattern of ER and Her2 expression in MDA-MB-453 and SK-BR-3 cell lines has been previously reported [30, 31] and provide a valid model for the study. MDA-MB-231 breast cancer cell line was used as ER- and Her2- controls. MDA-MB-453 was cultured in L15 medium (Thermo), 10% fetal bovine serum(FBS) (Gibco) and 1% penicillin/streptomycin (Life Technologies Inc). SK-BR-3 was cultured in DMEM medium (Gibco), 10% FBS and 1% penicillin/streptomycin. MDA-MB-231 were cultured in RPMI 1640 (Gibco) containing 10% FBS and 1% penicillin/streptomycin, under a humidified atmosphere with 5% CO2 at 37°C.

### Plasmid

The Lin28A siRNAs and Lin28A plasmid were chemically synthesized by Genechem (Shanghai, China). The Lin28A siRNA target sequences included: Lin28A siRNA#1 (si1)—5′-CTACTTTCGAGAGGAAGAA-3′, Lin28A siRNA#2 (si2)—5′-CTACAACTGTGGAGGTCTA-3′, Lin28A siRNA#3 (si3)—5′-TGGTGGAGTATTCTGTATT-3′. c-myc shRNA oligonucleotides for short hairpin RNAs were provided by Key Laboratory of Cancer Prevention and Therapy of Tianjin.

### Reverse transcription quantitative real-time polymerase chain reaction (qRT-PCR)

Briefly, total RNA was extracted using the Trizol reagent (Takara, Japan) according to the manufacturer's protocols. Reverse transcription was performed with the SuperScript RT kit (Takara, Japan), and the resultant cDNA templates were subjected to PCR amplification. To verify the level of cDNA integrity, β-actin expression was analyzed as the housekeeping gene in RT-PCR. The primer sets were 5′-TTGTCTTCTACCCTGCCCTCT-3′(forward) and 5′-GAACAAGGGATGGAGGGTTTT-3′ (reverse) for Lin28A, 5′-TTTGCCCATTGACTATTAC TTTCC-3′ (forward) and 5′-TTTCCCTTCAGCGGCTC TTT-3′(reverse) for AR, 5′-ATGCCCCTCAACGTT AGC-3′ (forward) and 5′-AGCTCGCTCTGCTGCTGC -3′ (reverse) for c-myc, 5′-AGCACTGGGGAGTCTTT GTG-3, (forward) and 5′-CTGAATGGGTCGCTTTTGTT -3′(reverse) for Her2, and 5′-AGCGAGCATCCCCCA AAGTT-3′(forward) and 5′-GGGCACGAAGGCTCA TCATT-3′(reverse) for β-actin. The specific reaction process and the calculation method were as mentioned previously [13]. Each sample was performed triplicate independently, and a mean value was used to calculate the mRNA levels.

### Western blot analysis

Cells were grown in 100-mm dishes and lysed in RIPA buffer and 1mM PMSF. The extracts were centrifuged and the supernatant fractions were collected for western blot analysis. Total cell lysates containing 30μg of protein were electrophoresed on a SDS-PAGE using precast 4 to 20% gradient Tris–glycine gels (Invitrogen,CA,USA) and then transferred to polyvinylidene fluoride(PVDF) membranes (Millipore,MA,USA). The membranes were blocked with 5% skim milk for 1 h at room temperature and then incubated with primary antibodies anti-Lin28A (ab75483; Abcam) at 1:200, anti-AR (D6F11; cell signaling) at 1:2000, anti-c-myc(ab32072; Abcam) at 1:5000, anti-GRO-α(ab86436; Abcam) at 0.5 μg/ml, anti-YB-1(ab76149; Abcam) at 1:1000, anti-E-cadherin (ab04722; Abcam) at 1:10000, anti-Vimentin (ab92457; Abcam) at 1:5000, anti-PCNA (ab92552; Abcam) at 1:5000, anti-Cyclin-E (ab33911; Abcam) at 1:2000, anti-Cleaved caspase-3 (ab32042; Abcam) at 1:1000, anti-Procaspase-3(ab32351; Abcam) at 1:5000, anti-Bcl-2 (ab32124; Abcam) at 1:1000, and anti-β-actin (KM9001T, Tianjin Sanjian) at 1:10000 4°C overnight. The membranes were then incubated with TBST containing horseradish peroxidase-labeled anti-rabbit IgG(1:5000) or horseradish peroxidase-labeled anti-mouse IgG (1:5000) and the chemiluminescence was detected by ECL. Western blot was done with triplicates for each sample.

### Chromatin immunoprecipitation (ChIP)

ChIP assay was performed according to instruction(Millipore). Chromatin extracts were precipitated with anti-c-myc(Abcam). ChIP assays were performed using primers spanning c-myc-binding sites in the AR promoter. Normal rabbit IgG was used as negative control. Data were normalized with respect to unprocessed lysates (input DNA). Input DNA quantification was performed by using 5ul of diluted (1/50) template DNA. The primer sets were 5′-GAGGGTTCCTAGAGCAAATGG-3′(forward) and 5′-CAGATGGGAGAGTGGGAGAG-3′(reverse) for AR promoter: c-myc binding site.

### Luciferase assays

MDA-MB-453 cells were seeded in culture medium on 24-well plates, serum starved for 24 h. MDA-MB-453 cells were transfected with pGL4-AR-prom-Luc reporters along with plasmids using the Lipofectamie 2000(Invitrogen, USA) as indicated in the figures. After 48 h of transfection, cell lysates were subjected to luciferase assays according to the Dual-Luciferase Reporter Assay protocol (Promega, Madison, WI). Each experiment was repeated in triplicates.

### MTT cell proliferation assay

The MTT assay was used to assess proliferative activity. Cells were seeded into 96-well plates at 5×10^3^ per well and incubated overnight under standard culture condition after Lin28A siRNA#2 and Lin28A plasmid transfection. Following incubation for 0, 24, 48 and 72h, 20ul of MTT solution was added to each well and the plates were incubated for another 4h at 37°C, and formazan crystals were dissolved with 150ul of Dimethyl Sulfoxide (DMSO). The absorbance of individual wells was read at 570 nm test wave length using a microplate reader (Bio-Rad Laboratories). Cell proliferation activities of transfected cells were determined by absorbance values.

### Invasion assays

Boyden chamber invasion assays were performed using the BioCoat Matrigel Invasion Chambers (Becton Dickinson Biosciences, Sparks, MD, USA). Briefly, the transfected cells were harvested and placed in the upper chamber (10^5^ cells per well) in serum-free medium, with complete medium in the lower chamber. After incubation at 37°C for 24 hours, the noninvading cells on the upper surface were scraped off, the invaded cells on the lower surface were fixed and stained. Finally, the number of invaded cells was counted under a microscope in nine random fields (×200).

### Clonogenic assays

A total of 1,000 cells were seeded in triplicate into six-well tissue culture plates and cultured at 37°C and 5% CO_2_ for 14 days. At the end of the experiment, colonies were fixed with methanol, stained with crystal violet, and counted.

### Detection of cell cycle by flow cytometry

The MDA-MB-453 cells transfected with Lin28A siRNA#2 and SK-BR-3 cells transfected with Lin28A were cultured for 48h and harvested. After digestion and centrifugation, the cells were fixed with 500ul of pre-cooling 70% ethanol at 4°C overnight. Then, 300ul of RNase containing propidium iodide (PI) staining solution was added to incubate at room temperature for 30 min. After washing with PBS, the absorbance was measured at 488 nm with flow cytometry.

### Detection of apoptosis by flow cytometry

The Annexin V-FITC binding assay was performed to determine the apoptosis rate of cells *in vitro*. Briefly, cells were seeded in a 6-well plate overnight, harvested 48 hours after treatment, and incubated with 5 microliter Annexin V-FITC and 5 microliter PI for 15 minutes at room temperature in the dark. Apoptosis was detected within 1 h in a flow cytometer. Each experiment was performed 3 times.

### *in vivo* experiments

Twenty female BALB/c nude mice of 4–6 weeks old and body weight of 20±2 g were housed in specific pathogenfree (SPF) conditions. The BALB/c nude mice were purchased from the Department of Laboratory Animal Science, Peking University Health Science Centre [license number: SCXK (Beijing) 2006–0008]. All the rats were maintained in an environ-mental-controlled room with clean air at 24°C with a 12h light/12h dark cycle. They were fed standard fodder and tap water. The rats were administered an subcutaneous injection of breast cancer cells at a dose of 2×10^6^ per case. Tumors were measured every 5 days, and tumor volume was calculated as: $\text{Tumor volume}=\frac{length^{\prime }\;width^{\prime }\;width}{2}$ [32].

All the rats were sacrificed after 30 days and the masses were resected. Livers and lungs were dissected immediately for further histopathological analysis. Liver and lung sections were stained with hematoxylin-eosin and examined blindly by two independent pathologists under light microscopy.

All the rat tumors were paraffin-embedded, cut into 4-um serial sections. The expression status of Lin28A, AR and Ki67 were determined by immunohistochemistry (IHC). IHC was performed using standard procedures. The expression level of Lin28A and AR was categorised as previously described [10]. Ki67 status was expressed in terms of percentage of positive cells, with a threshold of 20% of positive cells [33].

### Statistical analysis

The quantitative data were recorded as mean±SD and analyzed by one-way ANOVA and t-test. For all statistical analyses, the level of significance was set at p<0.05. The SPSS19.0 statistical programme was used for all statistical analyses. Each experiment consisted of at least three replicates per condition.
